# Chitosan-assisted hydrogen adsorption and reversibility of Ni-doped hierarchical carbon scaffolds

**DOI:** 10.1039/d4ra02687j

**Published:** 2024-06-14

**Authors:** Praphatsorn Plerdsranoy, Natthaporn Thaweelap, Suwabun Chirachanchai, Rapee Utke

**Affiliations:** a School of Chemistry, Institute of Science, Suranaree University of Technology Nakhon Ratchasima 30000 Thailand rapee.g@sut.ac.th; b Center of Excellence in Bioresources to Advanced Materials, The Petroleum and Petrochemical College, Chulalongkorn University Bangkok 10330 Thailand suwabun.c@chula.ac.th

## Abstract

We investigated the effects of chitosan (CS) on the hydrogen adsorption and reversibility of hierarchical carbon scaffold (HCS) loaded with Ni nanoparticles. As size-controllable, stabilizing, and shape-directing agents for the green synthesis of metal nanoparticles of CS, Ni nanoparticles with uniform distribution and shape are deposited onto HCS. The latter results in the superior specific surface area of Ni nanoparticles for hydrogen chemisorption. The best hydrogen adsorption capacities at room temperature under 20–70 bar H_2_ of 0.5–1.70 wt% H_2_ were obtained from 10 wt% Ni-doped HCS–CS. Although macropores of the HCS collapsed upon cycling due to hydrogen pressure applied during adsorption, average hydrogen capacities of 1.17 ± 0.05 wt% H_2_ (*T* = 25 °C and *p*(H_2_) = 50 bar) were maintained for 14 cycles. This is because not only uniform distribution and shape of Ni nanoparticles and microporous structures of the HCS were preserved upon cycling but also the interactions between Ni and heteroatoms (N and O) of the HCS and CS prevented particle agglomeration.

## Abstract 2

Investigation of how chitosan facilitates hydrogen sorption and reversibility of Ni-doped hierarchical carbon.

## Graphical abstract

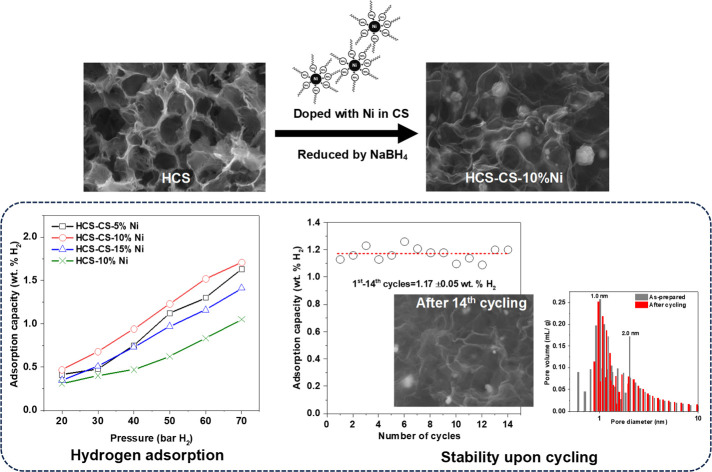

## 1. Introduction

Porous carbons with high specific surface area and porosity have drawn significant attention for several energy and environment-related applications owing to their remarkable properties of being lightweight, large surface area, and good thermal and chemical stability.^1–7^ In terms of energy storage, porous carbons have been intensively developed and utilized in batteries, supercapacitors, and hydrogen storage. For the latter application, weak van der Waals interactions between porous carbons and hydrogen with a low adsorption enthalpy of 4–10 kJ mol^−1^ hinder practical application since cryogenic temperature and high hydrogen pressure are required to store reasonable hydrogen capacities.^7,8^ Computational and experimental studies have reported that the ideal binding enthalpies of porous materials (15–30 kJ mol^−1^)^8,9^ for storing hydrogen at room temperature could be obtained from the modification of adsorbent structures,^10,11^ Kubas interactions,^12,13^ and spillover.^14^ For porous carbons, two main approaches, including (i) introduction of functional groups or heteroatoms as active sites for hydrogen adsorption (*e.g.*, amide, hydroxyl, acryl chloride, N, B, and S)^15–19^, and (ii) decoration with transition metal nanoparticles (*e.g.*, Mn, Fe, Ni, Co, Pt, Pd, and Cu)^14,20–24^ have been proposed to enhance hydrogen adsorption properties. Heteroatoms induce electron deficiency and enhance the polarity of carbon frameworks, leading to strong interaction between hydrogen and adsorbents. In addition, they strengthen the interaction between metal nanoparticles and carbon supports, resulting in good distribution of metal nanoparticles and inhibiting particle agglomeration and/or sintering upon cycling. These factors increase the reactive surface area of metal nanoparticles and promote hydrogen dissociative adsorption and spillover, facilitating hydrogen adsorption capacities and reversibility.^25–28^

Recently, our group investigated the hydrogen adsorption performances and mechanisms of Ni-doped activated carbon nanofibers (ACNFs)^28^ and hierarchical carbon scaffolds (HCSs)^27,29^ at ambient temperature. The mechanisms included the physisorption of hydrogen molecules in micropores as well as spillover and dissociative chemisorption on Ni nanoparticles. Strong interactions between Ni and heteroatoms of ACNFs and HCSs led to the uniform distribution of Ni nanoparticles. The latter enhanced the reactive surface area for hydrogen adsorption and prevented particle agglomeration upon cycling. However, the aggregation of Ni particles and significant pore blocking of ACNFs and HCSs were observed when Ni-loading contents were more than 5 wt%. This considerably reduced hydrogen adsorption capacities.^27,28^ The idea of utilizing chitosan as a stabilizing agent for metal nanoparticles is proposed to obtain greater Ni-loading contents with uniform distribution. Chitosan covering metal nanoparticles provided a steric barrier with a positive charge density, leading to strong electrostatic interactions among metal nanoparticles.^30^ This resulted in the formation of homogeneous and well-distributed metal nanoparticles (*e.g.*, Ag, Pt, Pd, Ni, and Fe/Ni nanoparticles).^31–34^ In the present work, 5–15 wt% Ni was deposited onto HCS using chitosan as the nanoparticle stabilizer. Greater Ni-loading content up to 10 wt% with uniform particle distribution and shape was obtained. Texture parameters, chemical compositions, morphology, and cycling stability of Ni-doped HCS samples with and without chitosan are characterized. The augmentation of chitosan for hydrogen adsorption performance and reversibility of Ni-doped HCS are investigated.

## 2. Experiments

### 2.1 Sample preparation

**Fig. 1:**
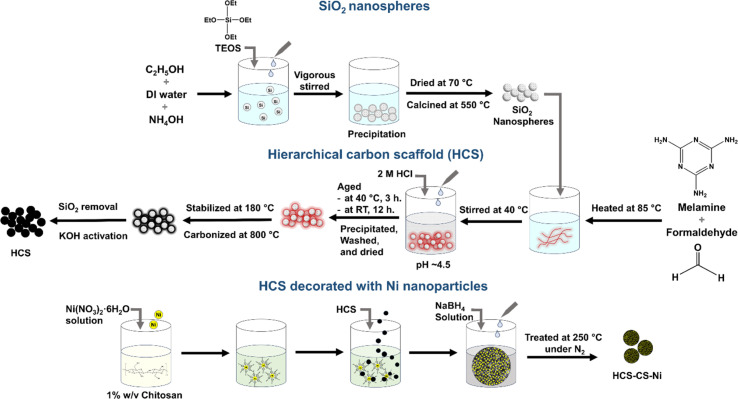
Schematic diagram for the syntheses of SiO_2_ nanospheres, HCS, and HCS decorated with Ni nanoparticles.

The syntheses of SiO_2_ nanospheres, hierarchical carbon scaffold (HCS), and HCS decorated with Ni nanoparticles are summarized in Fig. 1. HCS was prepared using melamine–formaldehyde resin and silica (SiO_2_) nanospheres as the carbon precursor and porous template, respectively.^27,35^ The mixture of absolute ethanol (292.50 mL, Merk), distilled water (50.00 mL), and ammonia solution (16.00 mL, 30% analytical grade, Carlo Erba) was stirred at room temperature for 20 min. Tetraethyl orthosilicate (25.00 mL, 98%, Acros Organics) was slowly dropped into the mixture, stirred vigorously for 1 h, left at room temperature for 12 h, and dried at 70 °C for 3 h. The obtained sample was calcined at 550 °C for 6 h, neutralized using 1.0 M HCl, washed with distilled water until reaching neutral pH, and dried at 80 °C overnight to obtain SiO_2_ nanospheres. Melamine (25.0056 g, 99%, Acros Organics), distilled water (62.50 mL), and formaldehyde (50.00 mL, 37% w/v, Carlo Erba) were stirred at 85 °C until a clear solution was obtained. SiO_2_ nanospheres (15.0025 g) well dispersed in 300.0 mL distilled water were added into the polymer solution and stirred at 40 °C for 1 h. The pH of the mixture was adjusted to ∼4.5 using 2.0 M HCl and aged at 40 °C for 3 h and at room temperature for 12 h. The white precipitate was separated from the solution, washed with distilled water and ethanol, and dried at 60 °C, overnight. The obtained sample was stabilized at 180 °C in air for 24 h and carbonized under N_2_ atmosphere at 800 °C for 2 h. SiO_2_ nanospheres were removed by soaking in 7.5 M HF at room temperature for 24 h. The obtained carbon scaffold was filtered, washed with distilled water and ethanol, and dried at 180 °C for 12 h. Activation was performed by immersing the carbon scaffold into 5.4 M KOH at 80 °C for 2 h, drying at room temperature for 24 h, and sintering at 800 °C under an N_2_ atmosphere for 30 min to achieve hierarchical carbon scaffold, denoted as HCS.

HCS decorated with Ni nanoparticles was prepared by the liquid-phase reduction method using sodium borohydride (NaBH_4_) and nickel(ii) nitrate hexahydrate (Ni(NO_3_)_2_·6H_2_O (99%, Acros Organics)) as a reducing agent and Ni source, respectively. Chitosan (CS, 1.00 g, *M*_w_ = 300–400 kDa, BIO21, Thailand) was dissolved in 0.1 M HCl (100.00 mL). HCS was dispersed into CS solution under a 1 : 1 weight ratio. Ni(NO_3_)_2_·6H_2_O solutions in DI water in accordance with the Ni-loading contents of 5, 10, and 15 wt% were stirred with HCS–CS mixture at room temperature for 1 h. NaBH_4_ solution in pre-cooled NaOH (0.13 M) of ∼25.00 mL was dropped slowly into Ni(NO_3_)_2_–HCS–CS mixtures and stirred vigorously for 30 min. All samples were dried at room temperature overnight, washed with DI water, and treated at 250 °C under an N_2_ atmosphere for 2 h to achieve HCS–CS loaded with 5, 10 and 15 wt% Ni, denoted as HCS–CS-5% Ni, HCS–CS-10% Ni, and HCS–CS-15% Ni, respectively. For comparison, HCS loaded with 10 wt% Ni was prepared. HCS (0.57 g) dispersed in 0.1 M HCl (50.00 mL) was stirred at room temperature for 1 h with Ni(NO_3_)_2_·6H_2_O solution, corresponding to 10 wt% Ni-loading. NaBH_4_ solution (∼25.00 mL) was dropped slowly into Ni(NO_3_)_2_–HCS mixture and stirred vigorously for 30 min. The obtained powder was dried at room temperature overnight, washed with DI water, and treated at 250 °C under an N_2_ atmosphere for 2 h to achieve HCS decorated with 10 wt% Ni, denoted as HCS-10% Ni.

### 2.2 Characterization

Surface area, pore size, and pore volume were investigated by N_2_ physisorption measurements using a BELSORP-mini II surface area and pore size analyzer, Bel-Japan. Prior to the measurements, the sample was degassed under vacuum at 250 °C overnight. All samples were characterized with full adsorption and desorption isotherms in the pressure range of 0–1 *p*/*p*_0_ at liquid nitrogen temperature with N_2_ gas as an adsorbent. The measurement was programed to continuously change the *p*/*p*_0_ ratio to 1 for adsorption, and to 0 for desorption. Data were analyzed by the *t*-plot method,^36,37^ the Brunner Emmett Teller (BET) method,^38^ and Barret Joyner Halenda (BJH) method.^39^ The highest point of the isotherm measurements (*p*/*p*_0_ ∼ 1) was used to calculate the total volume of the sample. Powder X-ray diffraction (PXRD) was carried out using a Bruker D2PHASER with a Cu K_α_ radiation (*λ* = 0.15406 nm). The diffraction patterns were collected in the 2*θ* range and the scanning step of 20–80° and 0.02° s^−1^. Morphology was studied by scanning electron microscopy (SEM) using an Auriga from Zeiss, Germany. The powder sample was deposited onto the sample holder using silver glue in *n*-butyl acetate. Regarding the electrical conductivity of HCS surface, coating with electron conductive elements (*e.g.*, Au) was not applied. XPS spectra were recorded using a Kratos Axis Ultra DLD (Kratos Analytical, U.K.) with monochromatic Al K_α_ X-rays (1486.69 eV). The binding energy was calibrated with respect to the C 1s peak at 284.8 eV and all spectra were fitted using MagicPlot program.

H_2_ adsorption/desorption measurements were carried out using a test station assembled with a controlling program developed using LabView® software.^27–29^ The powder sample (200–500 mg) was packed in a high-pressure sample cell (316SS, Swagelok) under N_2_ atmosphere in the glove box. Pressure transducers with an operating range of 0–100 bar absolute (Kistler type 4260A) were used to measure the sample and the reference pressures during the experiments. Two K-type thermocouples (−250 to 1300 °C, SL heater) were attached to the sample holder and the furnace for tracking the temperature during ad/desorption. The sample was degassed at 200 °C for 3 h under vacuum. Hydrogen adsorption was done at 25 °C under 20–70 bar H_2_ for 5 h, while desorption was carried out at 50 °C by releasing hydrogen gas from the sample and reference cells through a mass flow controller (MFC, a Bronkhorst EL-FLOW high-pressure model F-221M-RAD-22-V) with an operating flow rate of 0–0.1 standard L min^−1^ (SLM). The volume of the desorbed hydrogen (standard liter, SL) was obtained by integrating the area of the plot of hydrogen mass flow rate (SLM) *versus* time (min). The hydrogen content released from the sample cell subtracted from that from the reference cell was used to calculate the hydrogen storage capacity *via* the following equations.
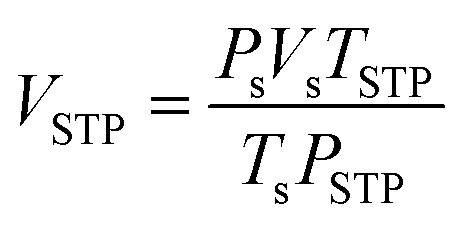

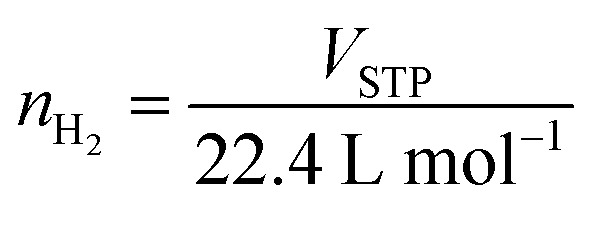

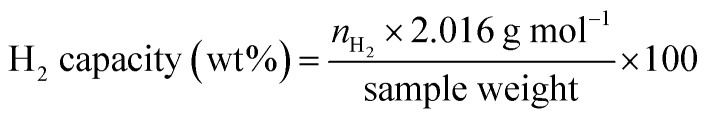
where *V*_STP_ (L) and *V*_s_ (SL) are volumes of hydrogen gas at standard temperature and pressure condition (STP, *T*_STP_ = 273.15 K and *P*_STP_ = 1.0133 bar) and at the standard condition of MFC (*T*_s_ = 294.95 K and *P*_s_ = 1.0167 bar), respectively. *n*_H_2__ (mol) is hydrogen moles and the standard molar volume is 22.4 L mol^−1^.

## 3. Results and discussion

**Fig. 2:**
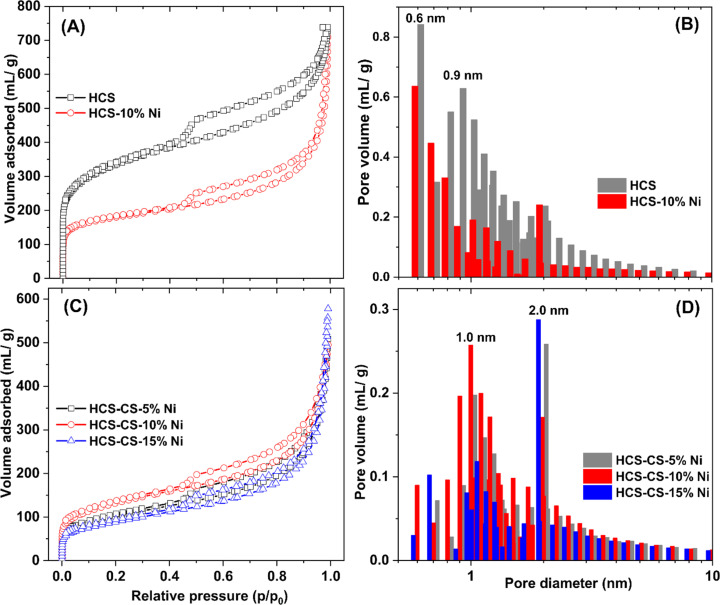
N_2_ adsorption isotherms (A and C) and pore size distribution (B and D) of the as-prepared samples.

**Fig. 3:**
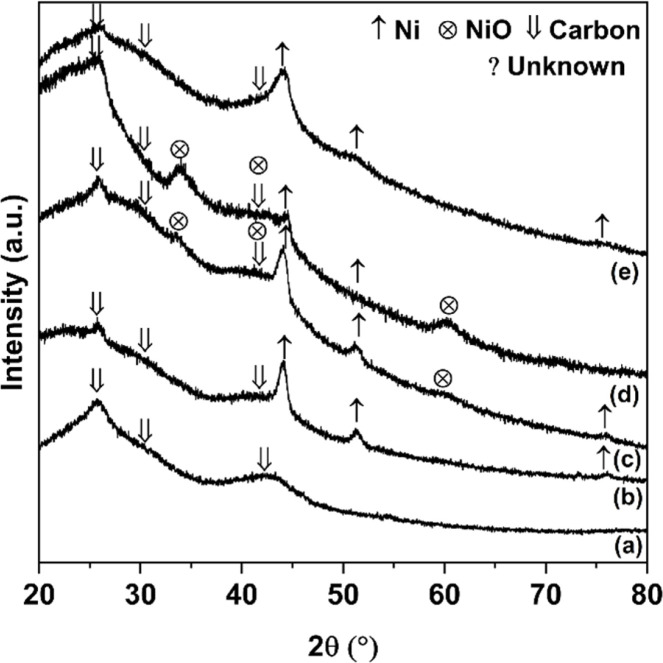
PXRD patterns of as-prepared samples of HCS (a), HCS–CS-5% Ni (b), HCS–CS-10% Ni (c), HCS–CS-15% Ni (d), and HCS-10% Ni (e).

Texture parameters and adsorption isotherms of HCS, HCS-10% Ni, and HCS–CS-Ni were characterized by the N_2_ physisorption technique. According to IUPAC classification, the powder samples of HCS, HCS-10% Ni, and HCS–CS-Ni show type IV isotherm with its feature of hysteresis loop (Fig. 2(A) and (C)), suggesting mesopores with good pore connectivity.^27,40,41^ The initial part of type IV isotherm follows the same path as the corresponding part of type II isotherm. The latter indicates the combined micro-/mesoporous adsorbents.^42^ These suggest the hierarchical porous structure with mixed micro-, meso- and macropores. Pore size distribution results reveal that the maximum pore volume of HCS is at the sizes of 0.6 and 0.9 nm, while that of HCS-10% Ni is at 0.6 nm (Fig. 2(B)). By doping with 10 wt% Ni, the pore size distribution of HCS at 0.6 nm is likely maintained, but the volume of the larger pores (0.9 nm) decreases significantly. For HCS–CS-Ni, the pore size distributions of HCS–CS-5% Ni and HCS–CS-15% Ni are comparable with the maximum pore volume at ∼2.0 nm, while that of HCS–CS-10% Ni is smaller ∼1.0 nm (Fig. 2(D)). Majority of the porous structures found in all samples are in the range of micropores (radius < 2 nm). In small pores, simultaneous interaction between hydrogen molecules and multiple pore walls of the adsorbents increases hydrogen adsorption enthalpy and storage capacities.^43,44^ From Table 1, the specific surface area (*S*_BET_) and the total volume (*V*_tot_) of HCS are 1173.8 m^2^ g^−1^ and 1.14 cm^3^ g^−1^, respectively, while those of HCS-10% Ni are reduced to 645.6 m^2^ g^−1^ and 1.07 cm^3^ g^−1^, respectively. For HCS–CS-Ni, *S*_BET_ and *V*_tot_ are further decreased to 316.9–464.1 m^2^ g^−1^ and 0.76–0.88 cm^3^ g^−1^, respectively (Table 1). The inferior *S*_BET_ and *V*_tot_ of HCS-10% Ni and HCS–CS-Ni compared to those of HCS are due to pore blocking of the agglomerated Ni particles and chitosan covering all over HCS. Chemical compositions of HCS–CS-Ni and HCS-10% Ni are characterized by the PXRD technique. From Fig. 3, all the as-prepared samples show comparable diffraction peaks of Ni and carbon (HCS). It should be noted that the diffraction of Ni in the PXRD spectrum HCS-10% Ni (Fig. 3(e)) is broader than that of HCS–CS-10% Ni (Fig. 3(c)). This suggests smaller particle sizes and/or greater amorphous state of Ni nanoparticles in HCS-10% Ni with respect to HCS–CS-10% Ni. The signal of NiO according to oxidation of Ni with oxygen and/or humidity during the experiments is detected in the PXRD pattern of HCS–CS-10% Ni and HCS–CS-15% Ni (Fig. 3(c) and (d)).

**Table 1:** Texture parameters of HCS-10% Ni and HCS–CS-Ni (5–15 wt%) in the as-prepared state and HCS–CS-10% Ni after cycling

Samples	*S*_BET_ (m_2_ g^−1^)	*V*_micro_ (cm^3^ g^−1^)	*V*_meso_ (cm^3^ g^−1^)	*V*_tot_ (cm^3^ g^−1^)
**As-prepared samples**				
HCS	1173.8	0.12	1.02	1.14
HCS-10% Ni	645.6	0.16	0.91	1.07
HCS–CS-5% Ni	362.6	0.01	0.76	0.77
HCS–CS-10% Ni	464.1	0.02	0.74	0.76
HCS–CS-15% Ni	316.9	0.02	0.86	0.88

**After cycling**				
HCS–CS-10% Ni	439.6	0.02	1.01	1.03

**Fig. 4:**
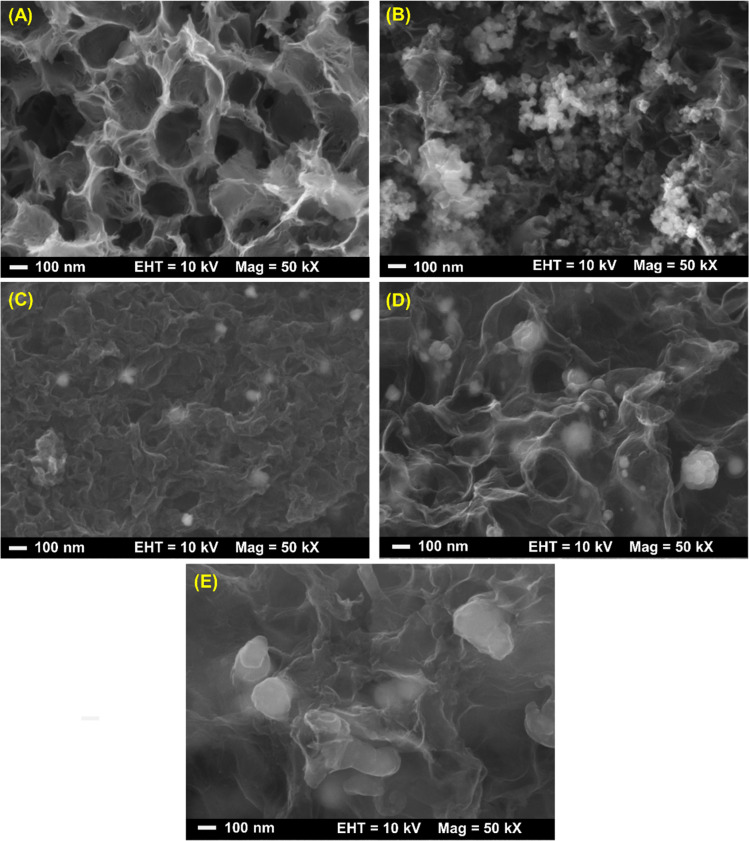
SEM images of HCS (A), HCS-10% Ni (B), HCS–CS-5% Ni (C), HCS–CS-10% Ni (D), and HCS–CS-15% Ni (E).

Furthermore, morphologies of HCS and all the as-prepared samples were studied by SEM technique. From Fig. 4(A), the SEM image of HCS shows the hierarchical porous structure of the mixed macro-, meso-, and micropores, corresponding to its adsorption isotherm shown in Fig. 2(A). For HCS-10% Ni, Ni nanoparticles with the sizes of ∼25 nm considerably aggregated, probably due to van der Waals interactions among metal surfaces (Fig. 4(B)). Such agglomeration of Ni nanoparticles leads to pore blocking and inferior *S*_BET_ and *V*_tot_ of HCS-10% Ni to those of HCS (Table 1). From Fig. 4(C), an SEM image of HCS–CS-5% Ni reveals a good distribution of Ni nanoparticles with the regular sizes of ∼50 nm. By increasing Ni-loading contents, particle sintering enhances with the sizes to 50–150 and 200–300 nm for HCS–CS-10% Ni and HCS–CS-15% Ni, respectively (Fig. 4(D) and (E)). The smaller particle sizes of Ni in HCS-10% Ni than those of HCS–CS-10% Ni are in accordance with the broader diffraction peaks of Ni detected in the PXRD pattern of HCS-10% Ni (Fig. 3(c) and (e)). Although the particle sizes of Ni in HCS–CS-Ni samples increase with Ni-loading contents, these Ni nanoparticles are well-distributed in HCS with a uniformly spherical shape, especially in HCS–CS-5% Ni and HCS–CS-10% Ni (Fig. 4(C)–(E)). The layer of chitosan covering HCS and Ni nanoparticles (Fig. 4(C)–(E)) is in accordance with the deficient *S*_BET_ and *V*_tot_ of HCS–CS-Ni (Table 1). However, the hierarchical porous structure of HCS in HCS–CS-Ni is preserved, confirmed by their adsorption isotherms (Fig. 2(C)). Chitosan was used as a size-controllable, stabilizing, and shape-directing agent for green syntheses of metal nanoparticles (*e.g.*, Ag, Pt, Pd, Ni, and Fe/Ni).^31–34^ Positively charged chitosan had strong electrostatic interaction with metal nuclei and inhibited the growth of metal nuclei during nanoparticle formation. Such interaction enhanced with chitosan concentration, leading to size reduction of metal nanoparticles.^30^ In our study, since all HCS–CS-Ni samples have a comparable HCS : CS weight ratio (1 : 1), the relative content of chitosan to Ni decreases with Ni-loading contents. Thus, the sizes of Ni nanoparticles increase with Ni-loading contents (Fig. 4(C)–(E)). For stabilizing and shape-directing effects, chitosan covering metal nanoparticles provides a steric barrier due to its positive charge density, resulting in uniform and well-distributed Ni nanoparticles.^30^ The latter agrees with the uniformly spherical shape and good distribution of Ni nanoparticles observed from HCS–CS-Ni (Fig. 4(C)–(E)).

**Fig. 5:**
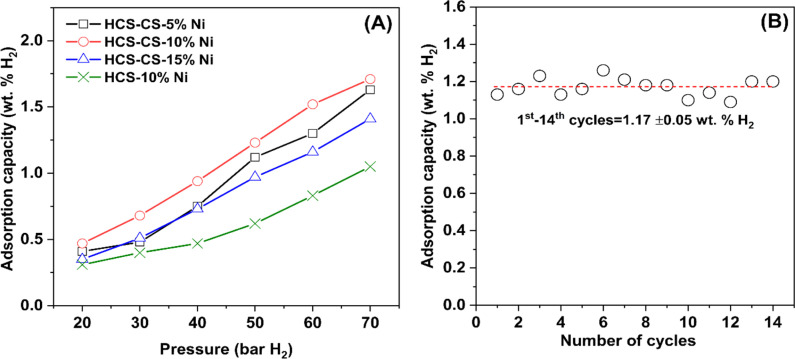
Hydrogen adsorption capacities at room temperature (∼25 °C) under 20–70 bar H_2_ of HCS–CS-Ni and HCS-10% Ni (A) and reversible hydrogen capacities at 25 °C under 50 bar H_2_ of HCS–CS-10% Ni (B).

Hydrogen adsorption capacities at room temperature (∼25 °C) under 20–70 bar H_2_ of HCS-10% Ni and HCS–CS-Ni were investigated. From Fig. 5(A), the adsorption capacities of HCS–CS-Ni increase to 0.50–1.70 wt% H_2_ with Ni-loading contents up to 10 wt%, whereas those of HCS–CS-15% Ni decrease to 0.35–1.40 wt% H_2_. These poor adsorption capacities at high Ni-loading content are explained by the reduction of active sites for hydrogen adsorption on the Ni surface due to particle growth, corresponding to the SEM image of HCS–CS-15% Ni (Fig. 4(E)). This result agrees with the previous reports of Ni-doped HCS^27^ and Ni-doped activated carbon nanofibers,^28^ in which hydrogen adsorption capacities were reduced with Ni loading due to particle agglomeration and/or sintering. According to the highest hydrogen adsorption capacities of HCS–CS-10% Ni, the experiments under similar temperature and pressure conditions (*T* ∼ 25 °C and *P* = 20–70 bar H_2_) of HCS doped with comparable Ni content of 10 wt% Ni (*i.e.*, HCS-10% Ni) were characterized for comparison. From Fig. 5(A), the hydrogen adsorption capacities of HCS-10% Ni (0.30–1.05 wt% H_2_) are inferior to those of HCS–CS-10% Ni. From our previous reports, experimental and computational studies confirmed that hydrogen adsorption at low temperatures (25–50 °C) of Ni-doped porous carbons (*e.g.*, HCS and activated carbon nanofibers) not only involved physisorption in micropores but also chemisorption and spillover on Ni nanoparticles.^27,28^ Considering physisorption, the optimum pore sizes of nanoporous carbons for hydrogen adsorption were in the range of 1.1–1.6 nm.^45,46^ The latter corresponds to the pore size distribution of HCS–CS-10% Ni, in which most pores have a diameter of ∼1.0 nm (Fig. 2(D)). In the case of chemisorption and spillover, the high reactive surface area of Ni nanoparticles for hydrogen adsorption was enhanced with the reduction of particle size and good particle distribution.^27,28^ Considering SEM images of HCS-10% Ni and HCS–CS-10% Ni (Fig. 4(B) and (D)), although the sizes of Ni nanoparticles in HCS-10% Ni (∼25 nm) are smaller than those of HCS–CS-10% Ni (50–100 nm), good dispersion and uniformly spherical shape of Ni nanoparticles in HCS–CS-10% Ni play important roles for enhancing hydrogen adsorption properties. This is likely due to the positive effects of chitosan as a size-controllable, stabilizing, and shape-directing agent for Ni nanoparticles.^30^ According to the highest hydrogen capacities of HCS–CS-10% Ni, its reversible adsorption capacities at ∼25 °C under constant pressure of 50 bar H_2_ were further studied. From Fig. 5(B), the average reversible hydrogen capacities upon 14 hydrogen release and uptake cycles of HCS–CS-10% Ni are 1.17 ± 0.05 wt% H_2_. These reversible capacities are comparable to those of 5 wt% Ni-doped activated carbon nanofibers (1.17 wt% H_2_),^28^ but they are inferior to those of HCS doped with 2–5 wt% Ni (1.25–1.50 wt% H_2_).^27,29^ This might be due to greater *S*_BET_ and *V*_tot_ of HCS doped with 2–5 wt% Ni (676.0 m^2^ g^−1^ and 0.36 cm^3^ g^−1^, respectively^27,29^) than those of HCS–CS-10% Ni (464.1 m^2^ g^−1^ and 0.76 cm^3^ g^−1^, respectively (Table 1)). The reduction of surface area and porosity leads to poor hydrogen physisorption into porous structure of HCS–CS-10% Ni.

**Fig. 6:**
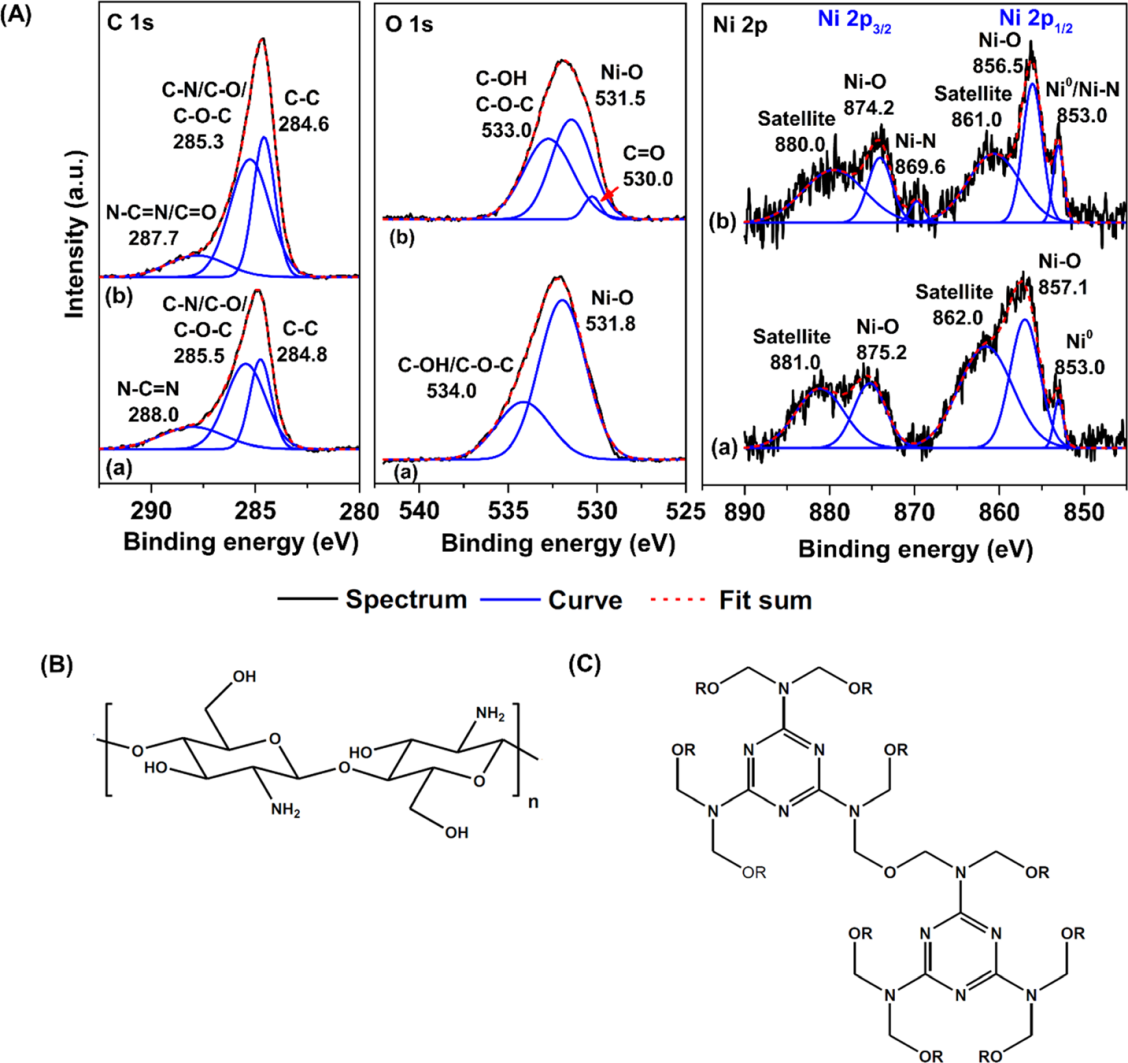
C 1s, O 1s, and Ni 2p XPS spectra (A) of HCS-10% Ni (a) and HCS–CS-10% Ni (b) as well as chemical structures of chitosan (B) and melamine–formaldehyde resin (C).

**Fig. 7:**
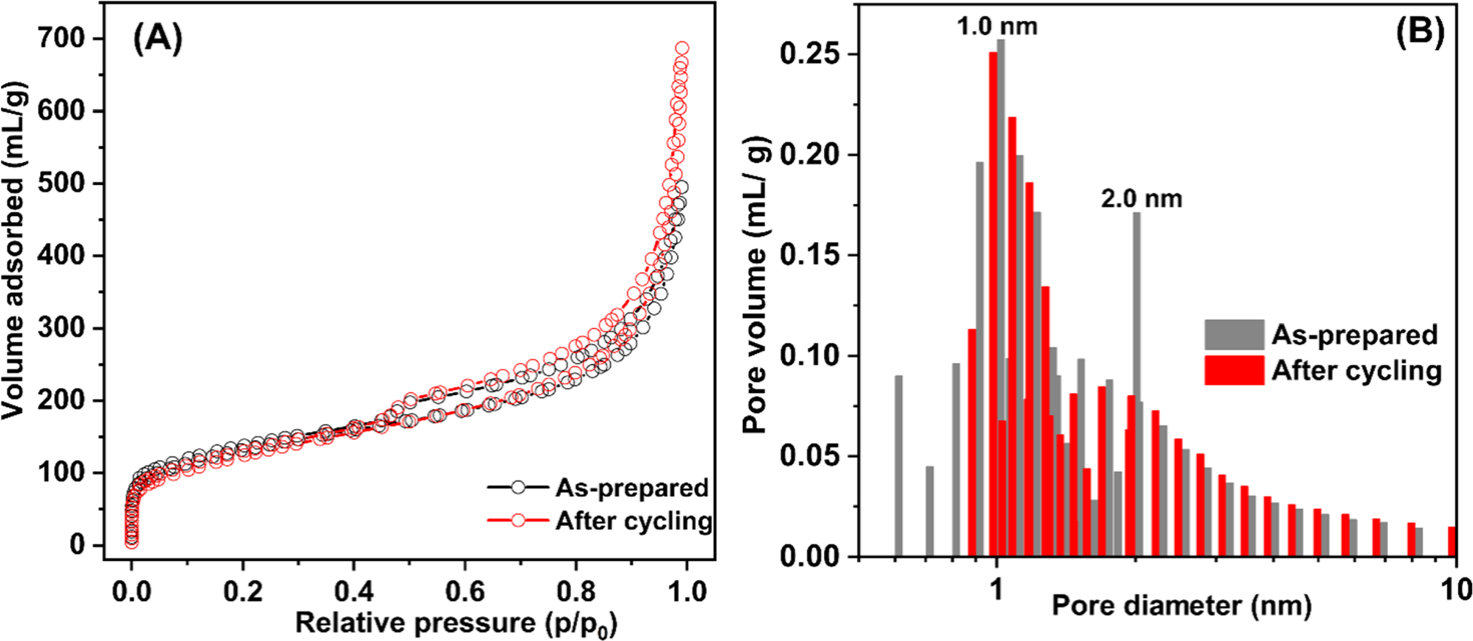
N_2_ adsorption isotherms (A) and pore size distribution (B) of HCS–CS-10% Ni in the as-prepared state and after cycling.

**Fig. 8:**
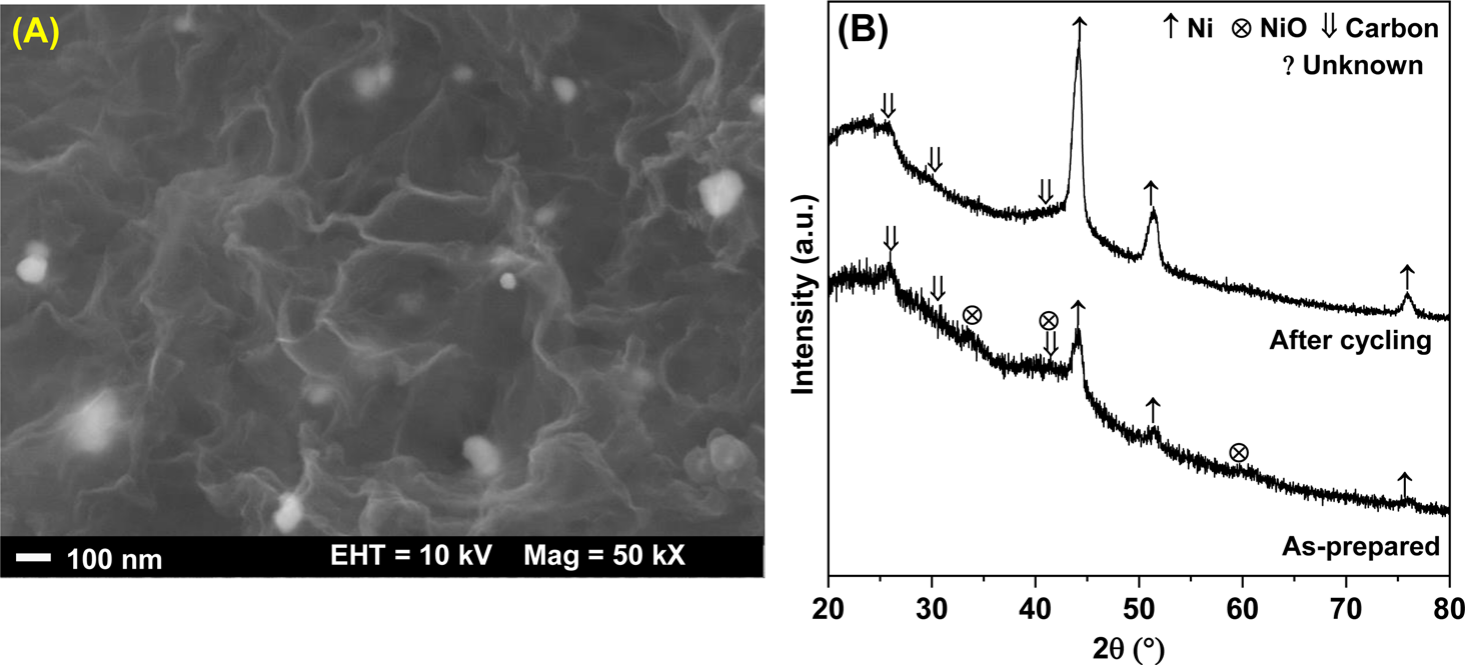
SEM image of HCS–CS-10% Ni after cycling (A) and PXRD patterns of HCS–CS-10% Ni in the as-prepared state and after cycling (B).

Furthermore, interactions between Ni nanoparticles and the supports (HCS or HCS–CS) of HCS-10% Ni and HCS–CS-10% Ni were characterized by the XPS technique. From Fig. 6(A), C 1s XPS spectra of HCS-10% Ni and HCS–CS-10% Ni show comparable characteristic peaks of C–C, C–N/C–O/C–O–C, and N–C=N at 284.6–284.8, 285.3–285.5, and 287.7–288.0 eV, respectively,^27,47,48^ while their O 1s XPS spectra reveal similar signals of C–OH/C–O–C at 533–534 eV. These results correspond to the functional groups of chitosan and melamine–formaldehyde resin used as carbon precursors during HCS synthesis (Fig. 6(B) and (C)). Moreover, the binding energy at 287.7 eV observed from C 1s XPS spectrum of HCS–CS-10% Ni is attributed to C=O of the carbonyl group,^49,50^ in accordance with the peak at 530.0 eV of O 1s XPS result (C=O)^51^ (Fig. 6(A)). Another role of chitosan is as a green reducing agent for synthesizing metal nanoparticles.^31–33,52^ Hydroxyl or glycosidic groups in the structure of chitosan provide the free electron to reduce metal ions to metal nanoparticles (*e.g.*, Ni^2+^ to Ni^0^ in this study) and transform to the carbonyl groups.^30^ The signals of the carbonyl group found in C 1s and O 1s XPS spectra of HCS–Cs-10% Ni confirm that chitosan not only acts as size-controllable, stabilizing, and shape-directing agents for Ni nanoparticles but also assists the reduction of Ni^2+^ to Ni^0^ during sample preparation. The latter leads to the efficient formation of metallic Ni nanoparticles and enhances active sites for hydrogen adsorption of HCS–CS-10% Ni. In the case of Ni 2p XPS results, both samples show the characteristic peaks in the ranges of 870–890 and 850–870 eV, corresponding to Ni 2p_3/2_ and Ni 2p_1/2_ spin–orbit levels, respectively (Fig. 6(A)). Ni 2p XPS spectra of HCS-10% Ni reveal the characteristic binding energies of Ni^0^ (853.0 eV) and Ni–O (857.1 and 875.2 eV), while that of HCS–CS-10% Ni contains not only Ni^0^ (853.0 eV) and Ni–O (856.5 and 874.2 eV) but also Ni–N (853.0 and 869.6 eV).^27,50,53^ The signal of Ni–O found in Ni 2p XPS spectra of HCS-10% Ni and HCS–CS-10% Ni agrees with the O 1s binding energies of Ni–O at 531.5–531.8 eV (Fig. 6(A)). These binding energies of Ni–N and Ni–O indicate the interaction of Ni nanoparticles with N or O atoms of HCS and chitosan, while that of Ni–O also relates to NiO from oxidation with oxygen and/or humidity during the experiments. It should be noted that HCS–CS-10% Ni shows not only Ni–O interaction comparable to HCS-10% Ni but also Ni–N. Since CS has high nitrogen content due to the presence of amine and acetamide in its structure,^54^ it has been used to synthesize several N-doped carbons, such as graphene-like N-doped carbon nanosheets,^55^ N-doped graphene aerogels, hierarchical porous carbon,^56^ and activated carbon.^57^ Due to the N-rich structure of CS, the interaction between Ni and the support (HCS–CS) through Ni–N is obtained. Heteroatoms (N and O in this study) introduced into porous carbons act as the anchoring sites for the deposition of metal atoms. This results in the uniform distribution of metal nanoparticles all over the supports, increasing reactive surface area for hydrogen adsorption and storage capacities.^25,27^ In addition, such interactions assist structural stability upon cycling of Ni nanoparticles dispersed on HCS, favouring hydrogen adsorption/desorption reversibility. Thus, significant interactions between Ni and heteroatoms of HCS and CS (O and N) observed in HCS–CS-10% Ni lead to superior hydrogen adsorption capacities and reversibility (Fig. 5). To confirm the stability in terms of texture parameters and morphology upon cycling, HCS–CS-10% Ni after 14 hydrogen ad/desorption cycles was characterized by N_2_ physisorption and SEM techniques. HCS–CS-10% Ni in the as-prepared state and after cycling show comparable adsorption isotherms and pore size distribution, *i.e.*, type IV isotherm with its feature of hysteresis loop and maximum pore volume at the size of ∼1.0 nm (Fig. 7). From Table 1, *S*_BET_ values of HCS–CS-10% Ni before and after cycling are comparable (∼440–464 m^2^ g^−1^). Porous structures with regular shape and size are observed from the as-prepared HCS–CS-10% Ni (Fig. 4(D)), but the uniform porous structure, especially macropores of HCS diminishes after cycling (Fig. 8(A)). Such changes in morphology and porous structure might be due to the high hydrogen pressure (50 bar H_2_) applied during the adsorption cycles. However, hydrogen adsorption capacities are preserved upon cycling (Fig. 5(B)). This indicates that the reversible hydrogen adsorption of HCS–CS-10% Ni does not significantly rely on this change in morphology, but it relates to (i) surface area and microporous structure and (ii) distribution, sizes, and shape of Ni nanoparticles. These properties of HCS–CS-10% Ni are maintained upon cycling, shown as N_2_ physisorption results (Fig. 7 and Table 1) and SEM image (Fig. 8(A)) of the sample after cycling. Moreover, chemical compositions of HCS–CS-10% Ni before and after cycling were investigated by the PXD technique. From Fig. 8(B), chemical compositions of HCS–CS-10% Ni are preserved upon cycling, *i.e.*, both samples before and after cycling show similar diffractions of Ni, NiO, and carbon.

## 4. Conclusions

Ni nanoparticles with uniform distribution and shape were loaded onto a hierarchical carbon scaffold (HCS) using chitosan (CS) as size-controllable, stabilizing, shape-directing, and reducing agents. During sample preparation, Ni^2+^ ions dispersed in CS solution were reduced to Ni^0^ by NaBH_4_ and CS. The obtained Ni^0^ was concurrently deposited onto HCS. Hydrogen adsorption capacities at room temperature under 20–70 bar H_2_ of Ni-doped HCS–CS increased to 0.50–1.70 wt% H_2_ with the Ni-loading content up to 10 wt%. However, the capacities of 15 wt% Ni-doped HCS–CS reduced to 0.35–1.40 wt% H_2_ due to the sintering of Ni particles. The best adsorption properties of 10 wt% Ni-doped HCS–CS was due to not only superior physisorption from high specific surface area (*S*_BET_ = 464.1 m^2^ g^−1^) and suitable pore size (∼1.0 nm) but also chemisorption due to good particle distribution and uniformly spherical shape of Ni nanoparticles. Upon 14 hydrogen ad/desorption cycles (*T* = 25 °C under 50 bar H_2_), hydrogen capacities of 10 wt% Ni-doped HCS–CS were preserved at an average of 1.17 ± 0.05 wt% H_2_. This was attributed to not only the fact that the microporous structure of HCS–CS (*S*_BET_ and pore size of 439.6 m^2^ g^−1^ and ∼1.0 nm, respectively) but also the sizes and shape of Ni nanoparticles were maintained. Besides, this cycling stability was explained by the strong interaction between Ni nanoparticles and heteroatoms of HCS–CS (N and O) preventing particle agglomeration upon cycling.

## Author contributions

Praphatsorn Plerdsranoy: conceptualization, sample preparation, characterizations, data analysis, and manuscript writing. Natthaporn Thaweelap: characterizations. Suwabun Chirachanchai: conceptualization, funding acquisition, resources, supervision, data analysis, manuscript writing, and reviewing and editing. Rapee Utke: conceptualization, supervision, funding acquisition, resources, data analysis, validation, manuscript writing, and reviewing and editing.

## Conflicts of interest

There are no conflicts to declare.

## Supplementary Material
